# Forge Ahead for Excellent Journals in Critical Care

**DOI:** 10.1007/s44231-022-00013-4

**Published:** 2022-09-22

**Authors:** Zhenxi He

**Affiliations:** Chinese Research Hospital Association, Beijing, China

As time flies by, 2022 is an extraordinary year for the development of intensive care medicine in China, which coincides with the first anniversary of the publication of *Intensive Care Research*. On behalf of Chinese Research Hospital Association and the editorial board of *Intensive Care Research*, I would like to take this opportunity to express my heartfelt thanks to the readers and writers who care for and support the journal. *Intensive Care Research* is co-sponsored by the First Affiliated Hospital of Zhengzhou University and Chinese Research Hospital Association, published in collaboration with Springer Nature. It aims to publish and deliver the latest expert guidelines/consensus on diagnosis, treatment, nursing, management of critical care medicine, case analysis, original research, review, etc. The first editorial board consists of 53 critical care medical experts from 6 countries. Over the past year, members of the editorial board have been forging ahead with their sincere cooperation and joint efforts, making a solid foundation of the journal and a good start towards an excellent critical care journal.

Chinese Research Hospital Association is a professional, academic, non-profit social organization composed of medical institutions with the functions of medical service, scientific research and clinical teaching aiming to explore and to build research hospitals, medical scientific research institutes, enterprises and institutions engaged in medical technology innovation and achievement transformation. The purpose of the society is to promote the integrated development of clinical and scientific research, to promote the continuous improvement of the level of clinical diagnosis and treatment, to accelerate the transformation of medical scientific and technological achievements, to promote the continuous generation of compound talents, and to promote the overall innovation of the hospital development model.

Since the theory and practice of establishing research-oriented hospital was put forward and implemented in 2004, it has gained a growing degree of recognition and consensus in the domestic medical and health field. Many managers, experts and professors regarded the construction of research-oriented hospital and discipline as a powerful starting point to promote hospital construction and discipline development. Over the past 9 years, the institute has become a national, all-field, think-tank-type authoritative medical organization with extensive influence with 139 branches, more than 300 clinical and scientific research hospital unit members and more than 21,000 expert members. Chinese Research Hospital Association has gathered leading figures in hospital management and influential experts and scholars at all levels, playing a positive role in promoting medical science and technology innovation and achievement transformation, strengthening the cultivation of interdisciplinary medical talents, establishing modern hospital management system and fighting against COVID-19. In the future, the association will further give full play to the role of think tanks, carry out forward-looking, targeted, continuous and sustainable policy research with the theme of promote the high-quality development of public hospitals, build the association into an internationally renowned and domestic first-class high-level professional organization with Chinese characteristics which can powerfully lead the construction of research-oriented hospital with extensive and in-depth academic exchanges and standardized institution, and make a contribution to promoting the high-quality development of public hospitals, deepening the reform of the medical and health system, and promoting the construction of healthy China at the same time.

As a new discipline, critical care medicine has been developing rapidly in recent years, attracting increasing attention from the medical circles and society. Since the pandemic outbreak of COVID-19, the importance and role of critical care medicine have been highlighted. *Intensive Care Research* has published a number of high-quality papers related to critical care medicine, timely and effectively spreading the latest scientific research achievements in critical care medicine, and has become an important front for international academic exchanges of critical care medicine.

Regarding the construction and development of the journal, I have the following suggestions and expectations: (1) Lead the hotspots of critical care medical research, and vigorously enhance the core competitiveness and attention of the journal; (2) Increase the sources of contributions, such as organizing high-level academic conferences, adding special issue columns, inviting contributions; (3) Spread the journal content through multi-channels. Articles published in the journal can be disseminated through the official website of Springer Nature, Google Scholar, WeChat official accounts, Twitter and other databases and platforms, as well as conferences to enhance the academic influence of the journal; (4) Strictly control the quality of papers to ensure quality firstly, which is fundamental; (5) Shorten the review cycle, such as expanding the reviewer team by increasing the number of young editors; (6) Cooperate with overseas co-editors to increase international dissemination and influence.

Looking forward to the future, *Intensive Care Research* will stay true to the original aspiration, forge ahead innovatively, actively lead the research hotspots of critical care medicine and strive to become an excellent critical medical journal as soon as possible!

